# Reply to “Reconsidering periosteal denervation: Anatomical redundancy and the limits of single-target interventions”

**DOI:** 10.1016/j.inpm.2025.100725

**Published:** 2025-12-24

**Authors:** John Tran, Brent Lanting, Zachary L. McCormick, Eldon Loh

**Affiliations:** a Division of Anatomy, Department of Surgery, University of Toronto, Toronto, Ontario, M5S 1A8, Canada; b Department of Physical Medicine and Rehabilitation, Parkwood Institute, London, Ontario, N6C 0A7, Canada; c Department of Orthopedic Surgery, University Hospital, Western University, London, N6A 5A5, Canada; d Department of Physical Medicine and Rehabilitation, University of Utah School of Medicine, Salt Lake City, 84108, USA

**Keywords:** Bone pain, Nutrient foramina, Articular nerves, Cutaneous nerve injury, Subchondral innervation, Knee osteoarthritis

Dear Editor,

We thank Dr. Sonawane for expressing interest in our recent study “Distribution of Epiphyseal Nutrient Foramina in the Distal Femur: Implications for Anterior Knee Joint Denervation” [1]. The innervation of the knee region is complex, owing to the different anatomical structures that comprise the knee which include tendons, ligaments, adipose tissue and bony articulations – each of which can serve as potential pain generator [2,3]. We agree that understanding the anatomy and the specific innervation of various component-structures of the knee is important to optimize specific treatment targets for individual patients depending on their suspected pathology.

In the context of our recent study [1], which focused on chronic knee joint pain related to osteoarthritis (OA), the “dominant nociceptive driver” or pain generator is likely subchondral bone-on-bone contact between the femur and tibia from cartilage degradation. As such, the extrinsic innervation that enters the nutrient foramina to supply subchondral bone should be the focus. Although nociceptive signals can derive from “motor, articular, cutaneous, and nutrient artery–associated fibers”, as noted by Dr. Sonawane, this is only relevant if these fibers are destined for the subchondral bone of the distal femur. For example, in a previous publication, we reported terminal branches of the nerve to vastus intermedius (motor classification) coursing through the vastus intermedius prior to entering the fascial plane superficial to the periosteum (Fig. 1A) [4]. These terminal fibers were described as articular branches terminating in the capsule and/or nutrient foramina, thereby making them amendable to periosteal-level interventions (without interrupting motor fibers) and particularly relevant targets when the subchondral bone is the dominant nociceptive driver.

**Fig. 1:**
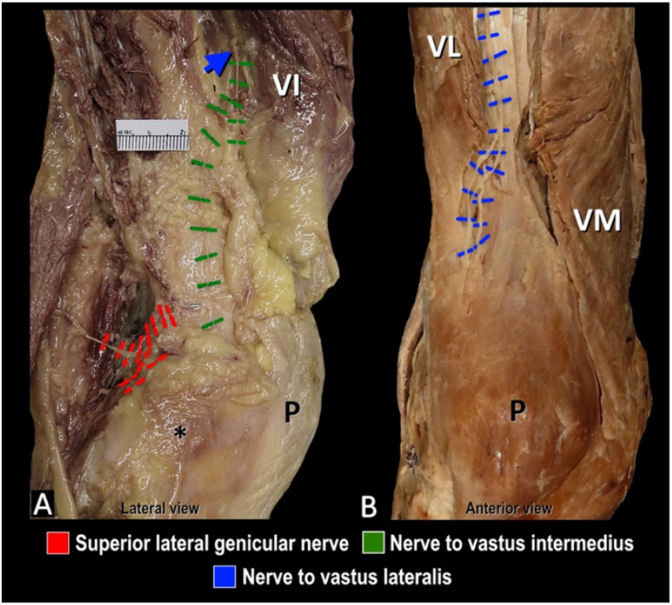
**Innervation of the knee.** A. Articular branch of the nerve to vastus intermedius coursing through the corresponding muscle (blue arrow) to enter fascial plane superficial to periosteum. B. Course of articular branches of nerve to vastus lateralis in intermuscular plane to supply the superior lateral aspect of the patella. Asterisk (*) indicates lateral femoral epicondyle; P, patella; VI, vastus intermedius; VL, vastus lateralis; VM, vastus medialis. (For interpretation of the references to colour in this figure legend, the reader is referred to the Web version of this article.)

We agree with Dr. Sonawane that comprehensive or complete analgesia may not be possible with a single target. In the past, we have advocated for the addition of supplemental lesions at each of the three classical landmarks to address the distribution and location of nerves as identified in cadaveric studies [4,5]. Additionally, chronic knee pain can arise from other structures besides the tibiofemoral joint. For example, patellofemoral OA, which has a reported prevalence of 25 % in individuals >20 years of age [6], may require targeting other articular branches such as the nerve to vastus lateralis (Fig. 1B). Similarly, pain from surgical injury to the infrapatellar branch of the saphenous nerve during total knee arthroplasty, would likely not be adequately addressed by procedures focused at the level of the periosteum. Targeting other potential knee pain generators requires further investigation and is beyond the scope of our current study [1].

In conclusion, our understanding of the anatomy pertaining to knee joint innervation is incomplete. Our recent osteology study on the nutrient foramina of the distal femur [1] represents the first of many studies that will help acquire a more complete understanding of the neuroanatomy of the knee. Whether complete knee denervation is necessary to adequately treat patients with chronic knee joint pain due to OA is unclear, though clinical experience demonstrates that complete analgesia is often possible with genicular nerve blocks using local anesthetic, indicating that if the territory of nerve disruption using radiofrequency ablation, chemical neurolysis, or other means can mirror the same distribution of local anesthetic spread, complete (durable) analgesia is conceivable with technical optimization of these procedures. Regardless, we advocate for a personalized, anatomically optimized, approach to treating patient-specific pain generators and additional anatomy and clinical studies are necessary to achieve this.

## Conflict of interests

John Tran, PhD has research grants from Avanos Medical and FUSMobile (paid directly to Lawson Research Institute), and also consultancies with Brixton Biosciences, and Merz Therapeutics (relationship ended).

Zachary L. McCormick, MD serves on the Board of Directors of the International Pain and Spine Intervention Society (IPSIS), has research grants from Avanos Medical, Boston Scientific, Relievant Medsystems, Saol Therapeutics, Spine Biopharma, SPR Therapeutics, Stratus Medical (paid directly to the University of Utah), and also consultancies with Avanos Medical, Saol Therapeutics, Stryker, and OrthoSon (relationships ended).

Eldon Loh, MD has research grants from Avanos Medical and FUSMobile (paid directly to Lawson Research Institute), and also is a consultant with Brixton Biosciences.

## References

[1] Tran J., Chung A.J., Bell I., Lanting B., McCormick Z.L., Loh E. (2025). Distribution of epiphyseal nutrient foramina in the distal femur: implications for anterior knee joint denervation. Interventional Pain Medicine.

[2] Benjamin M. (2004). Adipose tissue at entheses: the rheumatological implications of its distribution. A potential site of pain and stress dissipation?. Ann Rheum Dis.

[3] Ackermann P.W. (18 Jul. 2022). Tendon pain - what are the mechanisms behind it?. Scandinavian journal of pain.

[4] Tran J. (2020). Evaluation of nerve capture using classical landmarks for genicular nerve radiofrequency ablation: 3D cadaveric study. Reg Anesth Pain Med.

[5] McCormick Z.L., Cohen S.P., Walega D.R., Kohan L. (2021 Jun). Technical considerations for genicular nerve radiofrequency ablation: optimizing outcomes. Reg Anesth Pain Med.

[6] Kobayashi S. (2016). The prevalence of patellofemoral osteoarthritis: a systematic review and meta-analysis. Osteoarthr Cartil.

